# Ultralow-phase-noise millimetre-wave signal generator assisted with an electro-optics-modulator-based optical frequency comb

**DOI:** 10.1038/srep24621

**Published:** 2016-05-17

**Authors:** A. Ishizawa, T. Nishikawa, T. Goto, K. Hitachi, T. Sogawa, H. Gotoh

**Affiliations:** 1 NTT Basic Research Laboratories, Nippon Telegraph and Telephone Corporation, 3-1 Morinosato Wakamiya, Atsugi, Kanagawa 243-0198, Japan; 2 Tokyo Denki University, Department of Electrical and Electronic Engineering, 5 Senjyu-Asahi-cho, Adachi-ku, Tokyo 120-8551, Japan

## Abstract

Low-noise millimetre-wave signals are valuable for digital sampling systems, arbitrary waveform generation for ultra-wideband communications, and coherent radar systems. However, the phase noise of widely used conventional signal generators (SGs) will increase as the millimetre-wave frequency increases. Our goal has been to improve commercially available SGs so that they provide a low-phase-noise millimetre-wave signal with assistance from an electro-optics-modulator-based optical frequency comb (EOM-OFC). Here, we show that the phase noise can be greatly reduced by bridging the vast frequency difference between the gigahertz and terahertz ranges with an EOM-OFC. The EOM-OFC serves as a liaison that magnifies the phase noise of the SG. With the EOM-OFC used as a phase noise “booster” for a millimetre-wave signal, the phase noise of widely used SGs can be reduced at an arbitrary frequency *f* (6 ≦ *f* ≦ 72 GHz).

Low-noise millimetre-wave signals can benefit digital sampling systems^1^,^2^,^3^, arbitrary waveform generation for ultra-wideband communications^4^,^5^, and coherent radar systems^3^,^6^. Future wireless telecommunications with millimetre-wave signals will require increasingly high data-transmission efficiency. To meet this requirement, millimetre-wave signals with ultralow phase noise are essential. Several methods based on photonic technologies have been reported for generating millimetre waves, including whispering-gallery-mode parametric oscillators^7^, optical frequency division^8^,^9^,^10^, optoelectric oscillators^11^, on-chip Brillouin oscillators^12^, and optical reference cavities^13^. In those methods, since the millimetre-wave and microwave frequencies are fixed by using an optical cavity^7^,^8^,^9^,^11^,^12^,^13^, the output frequency of the millimetre-wave signals is not tunable. On the other hand, Li *et al.* determined the RF frequency based on the EOM frequency^10^. However, the wavelengths of the two lasers were locked to the free spectral range of a silica-high-Q disk with a high-finesse Fabry-Pérot cavity, which restricted the continuous variable range of the output frequency^10^.

To generate continuously tunable millimetre-wave and microwave signals with ultralow phase noise, we propose a new accessible solution that can greatly reduce the phase noise of widespread commercially available SGs with assistance from the EOM-OFC^14^,^15^,^16^. In our method, a mode-locked laser (MLL) is used as a “reference frequency ruler”. We can easily choose its nearest comb line with the selected EOM-OFC’s line regardless of SG 1’s frequency *f*_*m*_ and the mode number *k*. Although the phase noise in our method might not be reduced as much as in previous studies with a fixed frequency, we can achieve continuously tunable millimetre-wave and microwave frequency in a wide range because our method does not need an optical reference cavity, such as a silica high-Q disk or high-finesse Fabry-Pérot cavity. In addition, compared with other research-phase methods and commercially available ones for the generation of ultralow-phase-noise microwaves^8^,^17^, our method is extremely simple and the system is compact.

## EOM-OFC as a phase noise “booster”

**Figure 1:**
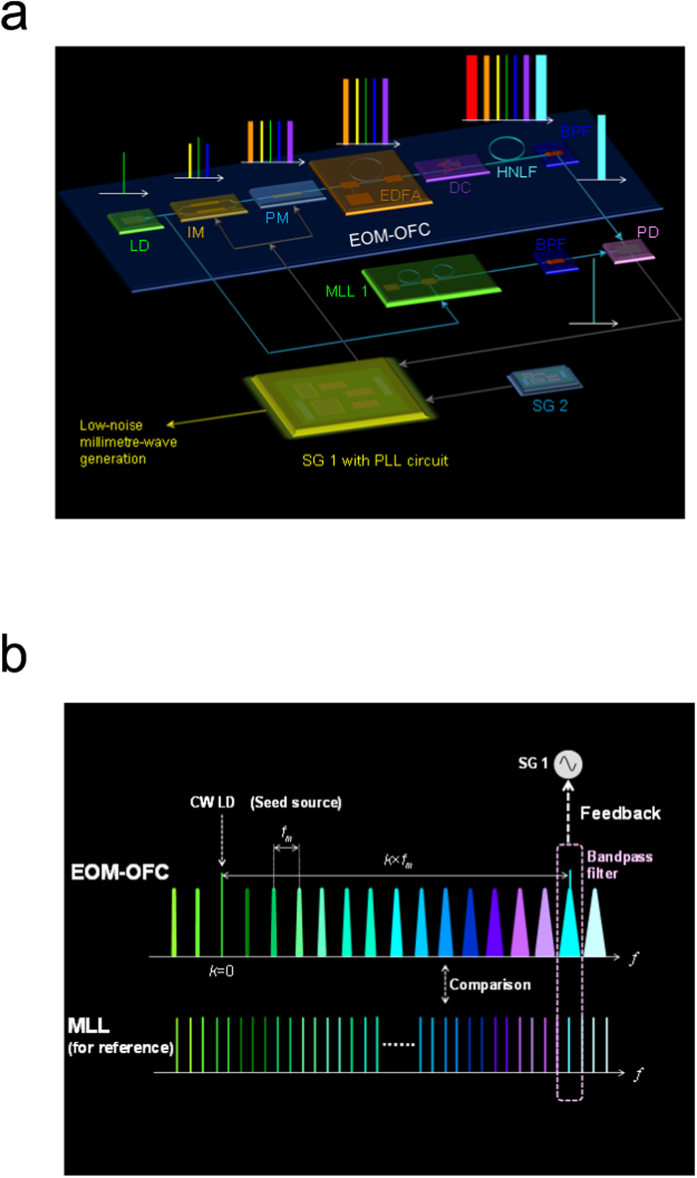
Our concept of low-phase-noise millimetre wave generation. (**a**) Experimental setup. SG: Signal generator. LD: Free-running laser diode. IM: Intensity modulator. PM: Phase modulator. EDFA: Er-doped fibre amplifier. DC: Dispersion controller. HNLF: Highly nonlinear fibre. BPF: Bandpass filter. MLL 1: Mode-locked laser with narrow linewidth. PD: Photodetector. (**b**) Spectral lines with the EOM-OFC and MLL 1.

Our concept of low-phase-noise millimetre-wave generation is shown in Fig.1a. We focus on the frequency difference between a few dozen gigahertz of the millimetre wave and a few hundred terahertz in the EOM-OFC^4^. The phase noise of the EOM-OFC mainly originates from that of SG 1’s driving phase/intensity modulators^18^ (see Supplementary Information). In addition, the phase noise of SG 1 is magnified as the comb mode number in the EOM-OFC increases. Our experimental setup is implemented to reduce the phase noise of SG 1 with a feedback circuit. SG 1 includes an yttrium iron garnet (YIG) oscillator with phase noise *φ*_1_(t). When a free-running continuous-wave laser diode (CW LD) is phase/intensity modulated by a sinusoidal signal with frequency *f*_m_, the *k*^th^ optical field of the EOM-OFC is expressed as


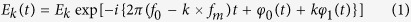


where *E_k_*, *f*_0_, and *φ*_0_(t) are the *k*^th^ optical field amplitude, the centre frequency of the EOM-OFC, and the phase noise of the CW LD, respectively (Fig. 1a,b). The comb mode number *k* 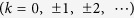 is defined as the number of comb modes from the CW LD frequency (comb mode number *k* = 0) as shown in Fig. 1b. As the frequency separation from the CW LD frequency increases, the phase noise of each comb mode of the EOM-OFC is magnified in proportion to the comb mode number^18^. On the other hand, MLL 1 has narrow comb lines as shown in Fig. 1a,b (also see Methods). The repetition rate of MLL 1 is 250 MHz. These narrow comb lines of 800 Hz at the full spectrum of MLL 1 are obtained by locking MLL 1 to the CW LD^19^. We use fibre-Bragg-grating filters with 0.1-nm bandwidth to filter the comb modes. An interference signal *I*_int_ between the frequencies of the nearest comb is generated by combining the EOM-OFC with MLL 1. SG 2 generates a sinusoidal RF signal *I*_ref_ at 60 MHz with a 90-degree phase shift for the interference signal *I*_int_ and is synchronized with a reference signal from a global positioning system (GPS). The phase difference between the interference signal *I*_int_ and the RF signal *I*_ref_ is detected with the phase detector inside SG 1. The low-frequency component is then selected with a lowpass filter. The YIG-oscillator-based VCO inside SG 1 adjusts the voltage so that the phase difference becomes zero (see Supplementary Information for details). Finally, *φ*_1_(t) is expressed as


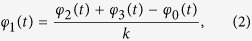


where *φ*_2_(t) and *φ*_3_(t) are the phase noises of MLL 1 and SG 2, respectively. From eq. (2), φ_1_(t) decreases as the reference comb mode number *k* increases. Our method can generate both millimetre waves and microwaves with low phase noise from SG 1 at an arbitrary frequency *f* (6 ≦ *f* ≦ 78 GHz) by using a frequency-variable VCO based on a monolithic microwave integrated circuit (MMIC). The YIG-oscillator-based VCO and the MMIC VCO are combined in order to achieve the frequency tunability. In the current experimental setup, SG 1 is continuously tunable at discrete ranges because the MMIC VCO cannot generate a signal in a wide tunable frequency range. However, our experimental results suggest that if we use an MMIC VCO with wider tunable frequency range, SG 1 will become continuously tunable over the entire range from 6 to 78 GHz.

## Phase-noise reduction of widely used conventional SGs

**Figure 2:**
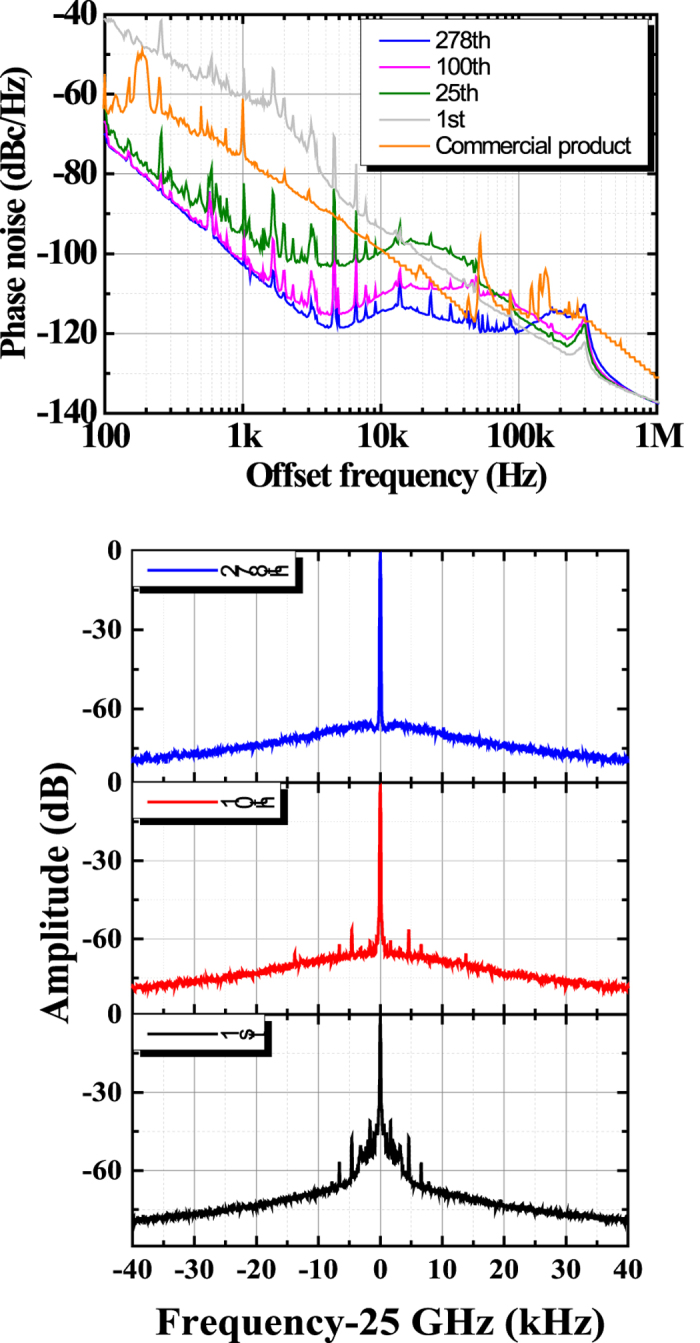
Dependence of experimental phase-noise spectra of the 25-GHz millimetre waves on the reference comb mode number. (**a**) Measured phase noise of the commercial product (orange trace) and of SG 1 feeding back with the 1^st^ mode (grey trace), 25^th^ mode (green trace), 100^th^ mode (pink trace), and 278^th^ mode (blue trace) of the EOM-OFC. (**b**) Measured millimetre-wave spectrum via SG 1 in the 1^st^ mode (black trace), 10^th^ mode (red trace), and 278^th^ mode (blue trace).

We experimentally demonstrated that *φ*_1_(t) at 25 GHz decreases as the reference comb mode number of the EOM-OFC increases, as shown in Fig. 2a. The 1^st^, 25^th^, 100^th^, and 278^th^ comb mode numbers correspond to wavelengths of 1553, 1556, 1573, and 1611 nm, respectively. The reference frequency of SG 2 is set at 60 MHz and used for phase-noise reduction. Since the feedback loop bandwidth is set at 100 kHz, *φ*_1_(t) can be reduced at an offset frequency of less than 100 kHz. When we feedback with the 1^st^ comb mode number, *φ*_1_(t) is larger than that of the commercially available product with the lowest phase noise. Since the comb mode number *k* (=1) is small, *φ*_1_(t) is not greatly reduced. With the current experimental setup, *φ*_1_(t) is lowest with the 278^th^ comb mode number because the intensity of supercontinuum (SC) generation decreases at larger comb mode numbers. Under our experimental conditions, the lowest *φ*_1_(t)’s values are −118 and −120 dBc/Hz at offset frequencies of 5 and 100 kHz, respectively. The phase noise we achieved with SG 1 is far lower than the lowest phase noise reported for commercially available SGs. Figure 2b shows the 25-GHz millimetre-wave spectrum of SG 1 generated by using each mode of the EOM-OFC, which was measured with a 100-Hz resolution bandwidth. We found that the linewidth becomes narrower as the comb mode number increases. The results in Fig. 2a,b are consistent. The spurious emission in Fig. 2b shows the frequency and intensity stability of the 25-GHz millimetre waves. Since the tuning sensitivity of the feedback circuit in SG 1 is adjusted at the 278^th^ comb mode number, the spurious emission of the 25-GHz millimetre-wave signal becomes lower as the comb mode number approaches the 278^th^ as shown in Fig. 2b.

## Low-phase-noise SGs over a wide frequency range with EOM-OFC

**Figure 3:**
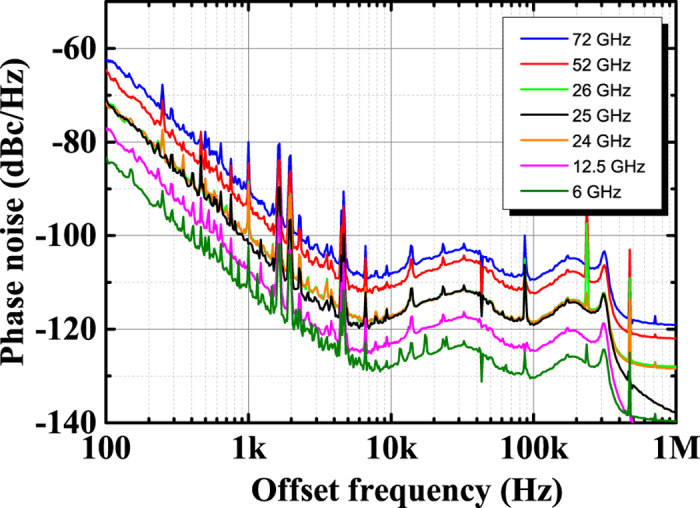
Experimental phase-noise spectra from the 6-GHz microwave to the 72-GHz millimetre wave with 278^th^ mode of the EOM-OFC. Measured phase noise at 6 (green trace), 12.5 (pink trace), 24 (orange trace), 25 (black trace), 26 (yellow-green trace), 52 (red trace), and 72 GHz (blue trace) output from SG 1 and fed back with the 278^th^ mode of the EOM-OFC.

Next, we performed measurements over a wide frequency range from 6 to 72 GHz. Figure 3 shows the phase noise spectra from 6 to 72 GHz with the 278^th^ mode of the EOM-OFC. We found that the phase noise of SG 1 from 6 to 72 GHz is reduced as the comb mode number in the EOM-OFC increases (not shown). Recently, there have not been any significant advances in ultralow-phase-noise SGs that use the traditional technique. The SG with a commercially based cryogenic sapphire oscillator^17^ has to be kept at a low temperature near 4 K with liquid helium. Our proposed technique makes it possible to reduce the phase noise of widely used conventional SGs without a cooling system. In addition, the low-phase-noise microwave frequency, such as that with an MLL in previous methods^8^,^9^, has been fixed because a laser cavity, which corresponds to the microwave frequency, is needed. On the other hand, our method is highly advantageous in that the SG is continuously variable over a wide frequency range from 6 to 72 GHz.

## Discussion

**Figure 4:**
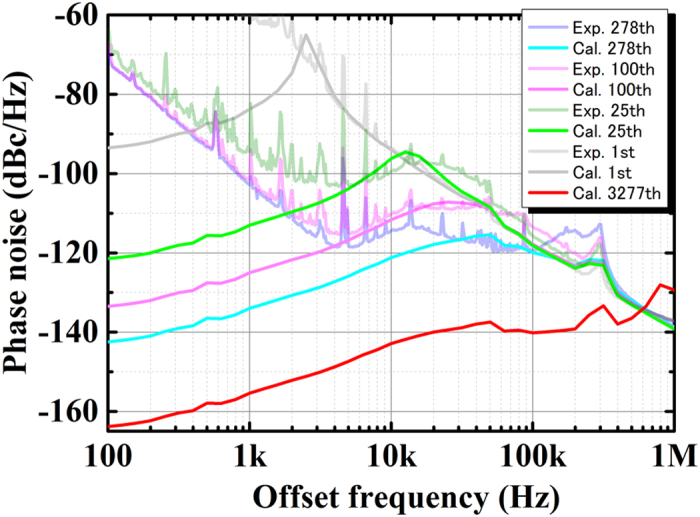
Simulated phase-noise spectra of 25-GHz millimetre waves. Calculated phase noise *φ*_1_(t) obtained when feeding back with the 1^st^ mode (grey trace), 25^th^ mode (green trace), 100^th^ mode (pink trace), 278^th^ mode (blue trace) of the EOM-OFC. As reference traces, the experimental phase-noise spectra (Fig. 2a) are shown by the faintly coloured curves.

We calculated phase noise *φ*_1_(t) using ADIsimPLL software^20^. The *φ*_1_(t) obtained after the feedback control is related to phase noise {*φ*_2_(t) − *φ*_0_(t)} because *φ*_2_(t) and *φ*_0_(t) are much larger than *φ*_3_ (t) [eq. (2)]. Phase noise {*φ*_2_(t) − *φ*_0_(t)} can be observed from the phase noise of the interference signal between MLL 1 and the CW LD (see Supplementary Information). We use the measured {*φ*_2_(t) − *φ*_0_(t)} for the calculation. The *φ*_1_(t) can be calculated by using certain parameters in the PLL synthesizer chip, the lowpass filter, and the reference frequency from SG 2. Then the tuning sensitivity of the YIG-oscillator-based VCO is adjusted because it depends on the reference comb mode number. Figure 4 shows that the calculated data are consistent with the experimental data at offset frequencies larger than 10 kHz. The *φ*_1_(t) is determined by the sum of {*φ*_2_(t) − *φ*_0_(t)} and the phase noise of the YIG-oscillator-based VCO. Actually, *φ*_1_(t) under our experimental conditions is larger than that calculated at a low offset frequency of less than 1 kHz. This is because of the slow drift of the centre frequency of the CW LD used in our experiment. When we use MLL 2 and control the carrier-envelope-offset (CEO) and repetition frequency, we can stabilize the centre frequency of the CW LD, although its linewidth increases. We investigated *φ*_1_(t) when both the centre frequency of the CW LD and the repetition frequency of MLL 2 were frequency stabilized (see Supplementary Information). The locking bandwidth is several kilohertz. Although *φ*_1_(t) with MLL 2 became larger than with MLL 1 because of the linewidth difference, we clearly found that *φ*_1_(t) at an offset frequency of less than 10 kHz decreases when the centre frequency of the CW LD is stabilized. This result indicates that we should be able to reduce *φ*_1_(t) at an offset frequency of less than 10 kHz (Fig. 2a) by stabilizing the centre frequency of the CW LD while maintaining a narrow linewidth. In addition, we should be able to estimate the lowest achievable *φ*_1_(t) in our method. The *φ*_1_(t) becomes lower as the reference comb mode number increases as shown in eq. (2). If we use an optical gate^21^, which can reduce the repetition rate with an intensity modulator and increase the peak intensity of the amplified pulse, we can generate a one-octave SC spectrum from 1090 to 2180 nm, where the shortest wavelength, 1090 nm, corresponds to the 3277^th^ mode. The calculated *φ*_1_(t) of the SG with the 3277^th^ mode is shown in Fig. 4. This result indicates the possibility of the phase noise’s becoming two orders of magnitude lower.

**Figure 5:**
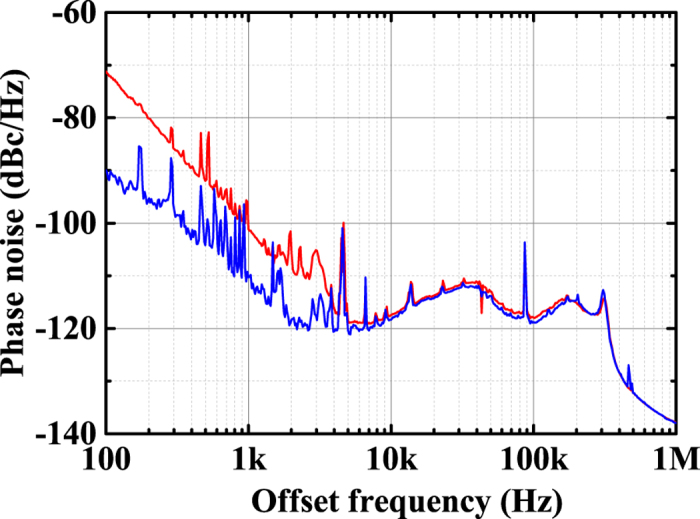
Phase noise *φ*_1_(t) of SG 1 at 25 GHz with two seed sources of different spectral linewidth. Measured phase noise *φ*_1_(t) of SG 1 with the 278^th^ mode of the EOM-OFC using the1-Hz linewidth laser, which is locked to a stable high-finesse cavity (blue), and using the free-running CW LD (red).

We found that the phase noise of widely used conventional SGs could be reduced if we use a more stable seed light source (see Supplementary Information, Fig. S7). To verify this experimental result, we compared a 1-Hz linewidth laser, which is locked to a stable high-finesse cavity, with the free-running CW LD as seed light sources. The centre wavelengths of the 1-Hz linewidth laser and the CW LD are 1542 and 1552 nm, respectively. As shown in Fig. 5, the phase noise *φ*_1_(t) of SG 1 with the 278^th^ mode of the EOM-OFC is reduced by using the more stable seed light source. The phase noise with the 1-Hz linewidth laser at the offset frequencies of 100 Hz and 1 kHz becomes 19 and 9 dB lower than with the CW LD, respectively. In addition, the RMS jitter at offset frequencies from 100 Hz to 100 kHz is reduced from 19.1 to 7.2 fs with the 1-Hz linewidth laser. It is found from the above experimental and calculated results that three parameters — the mode number of the EOM-OFC, the stabilization of the centre frequency of the seed source, and the narrow linewidth comb in MLL 1 — are essential for reducing the phase noise of widely used conventional SGs.

In summary, we have demonstrated that a millimetre wave with low phase noise can be generated from commercially available SGs by using an EOM-OFC and a feedback circuit. These results show that the EOM-OFC can work as a phase noise booster and a high-sensitivity detector for detecting the magnified phase noise of an SG in the optical frequency domain. This method can reduce the phase noise of not only an SG but also of a microwave generation source with a photonic approach. The capability of greatly reducing the phase noise of SGs simply by employing an EOM-OFC makes SGs suitable for applications requiring spectrally pure signals, such as digital sampling systems^1^,^2^,^3^, arbitrary waveform generation for ultra-wideband communications^4^,^5^, communications, and coherent radar systems^6^. In addition, 920- and 30-km-long optical fibre links have been reported recently^22^,^23^. Once we can easily use the highly stable laser from an optical fibre link as the seed source of the EOM-OFC, ultralow-phase-noise SGs with our method would become universal.

## Methods

### Narrow linewidth comb with a mode-locked laser

For this narrow linewidth comb, we employ MLL 1 with an EOM and a piezoelectric element inside the cavity. With a CW LD as a reference light source, all the combs within the full bandwidth of MLL 1 can be narrowed to the 800-Hz linewidth of the CW LD by using the EOM, the piezoelectric element in MLL 1, and the feedback circuit^19^,^24^. The CEO of MLL 1 is stabilized with an *f*-to-2*f* self-referencing interferometer, and the repetition rate is locked with the interference signal between the CW LD and MLL 1. Both the CEO and the repetition rate of MLL 1 are stabilized by using the same GPS signal as for SG 1 as the reference signal. Since the frequency of the CW LD is only temperature-controlled, the repetition rate slowly drifts. The comb mode from MLL 1 that is nearest to the *k*-mode comb of the EOM-OFC at the optical frequency is selected with an optical bandpass filter. MLL 1 can be replaced with a narrow linewidth CW LD. It is essential to use a narrow line spectrum.

### Feedback control of phase noise *φ*_1_(t)

We use an EOM-OFC with 25-GHz mode spacing and MLL 1. Two optical bandpass filters transmit each comb of the EOM-OFC and MLL 1. The beam is coupled into an InGaAs photodetector. From the signal, the heterodyne beat between the interference components yields a frequency difference *I*_int_(t). On the other hand, SG 2 generates a 60-MHz reference signal. The phase difference between *I*_int_(t) and the reference signal is then measured with the phase detector inside SG 1. The loop filter inside SG 1 uses the phase difference to calculate the frequency modulation. Finally, the voltage in the YIG-oscillator-based VCO inside SG 1 is adjusted to realize a zero phase difference.

## Additional Information

**How to cite this article**: Ishizawa, A. *et al.* Ultralow-phase-noise millimetre-wave signal generator assisted with an electro-optics-modulator-based optical frequency comb. *Sci. Rep.* **6**, 24621; doi: 10.1038/srep24621 (2016).

## Supplementary Material

Supplementary Information
