# Supplementary material for: Ultralow-phase-noise millimetre-wave signal generator assisted with an electro-optics-modulator-based optical frequency comb
Source: Sci Rep. 2016 May 17;6:24621. doi: 10.1038/srep24621 (PMC4869108; doi:10.1038/srep24621)
Supplement: Supplementary Information [file srep24621-s1.pdf]

Supplementary Information

Ultralow-noise millimetre-wave signal generator assisted with an electro-optics-modulator based optical frequency comb

A. Ishizawa*[,1], T. Nishikawa[2], T. Goto[1,2], K. Hitachi[1], T. Sogawa[1], and H. Gotoh[1]

[1]*NTT Basic Research Laboratories, Nippon Telegraph and Telephone Corporation, 3-1 Morinosato Wakamiya, Atsugi, Kanagawa 243-0198, Japan*

[2]*Tokyo Denki University, Department of Electrical and Electronic Engineering, 5 Senjyu-Asahi-cho, Adachi-ku, Tokyo 120-8551, Japan*

*To whom correspondence should be addressed. E-mail: ishizawa.atsushi@lab.ntt.co.jp

The supplementary information contains the following sections:

1. Block diagram of SG1

2. Our concept of low-noise millimetre-wave generation

3. Supercontinuum generation from an EOM-OFC

4. Measurement of phase noise {$\varphi_2(t) - \varphi_0(t)$}

5. Relationship between phase noise φ_1 (t) and CW LD frequency stabilization

1. Block diagram of SG1

Figure S1 shows a block diagram of SG 1. The YIG-oscillator-based VCO and the frequency-variable VCO based on the MMIC are combined in order to achieve frequency tunability. In our method, all SGs other than SG 1 are synchronized with the GPS reference signal (10 MHz). The YIG oscillator generates the microwave signal at 6.25 GHz. By frequency multiplying the microwave signal by four, a 25-GHz millimetre wave is generated. The phase noise φ_1 (t) of the 25-GHz output signal on the YIG oscillator without PLL feedback is shown in Fig. S2. The 25-GHz millimetre wave is delivered to the IM and PMs, and then the EOM-OFC with 25-GHz mode spacing is generated. By combining the EOM-OFC with MLL 1, an interference signal at 60 MHz is generated. The interference signal is connected to the 60-MHz PLL INPUT in Fig. S1. The Phase Detector determines the relative phase difference between the 60-MHz PLL INPUT and the 60-MHz signal of the SG 2, and then the phase-locked YIG oscillator generates the stabilized microwave signal. Figure S3 shows the phase noise φ_3 (t) of

SG 2 synchronized with the GPS reference signal, which is much lower than the phase noise $\varphi_2(t)$ of MLL 1. The MMIC-based VCO, which we used in this experiment, is frequency-variable from 24 to 26 GHz. Both the 25-GHz millimetre wave from the YIG oscillator and the 24- to 26-GHz millimetre wave from the MMIC-based VCO are delivered into the MIXER. The signal from the MIXER is generated at frequencies from 100 MHz to 1 GHz by using a lowpass filter. Next, a commercial SG (SG 3) is set, and it generates the signal at frequencies from 50 to 500 MHz. The Phase Detector determines the relative phase difference between the signals from the MIXER and SG 3 using a counter (N=2-10), and then the phase-locked MMIC-based VCO generates the millimetre signal with low phase noise at continuously variable frequencies from 24 to 26 GHz. The millimetre signal from the MMIC-based VCO is delivered into the frequency tripler, frequency doubler, and 1/4 frequency divider. Then, the millimetre wave and microwave at continuously variable frequencies from 72 to 78, 48 to 52, and 6 to 6.5 GHz are generated, respectively. In this setup, the millimetre wave and microwave from SG 1 are generated at discrete variable frequencies because the variable range of the currently used VCO is ±1 GHz. If an MMIC-based VCO with a wider variable range is used, the millimetre wave and microwave from SG 1 could be generated at the continuously variable frequencies over the entire range from 6 to 78

GHz.

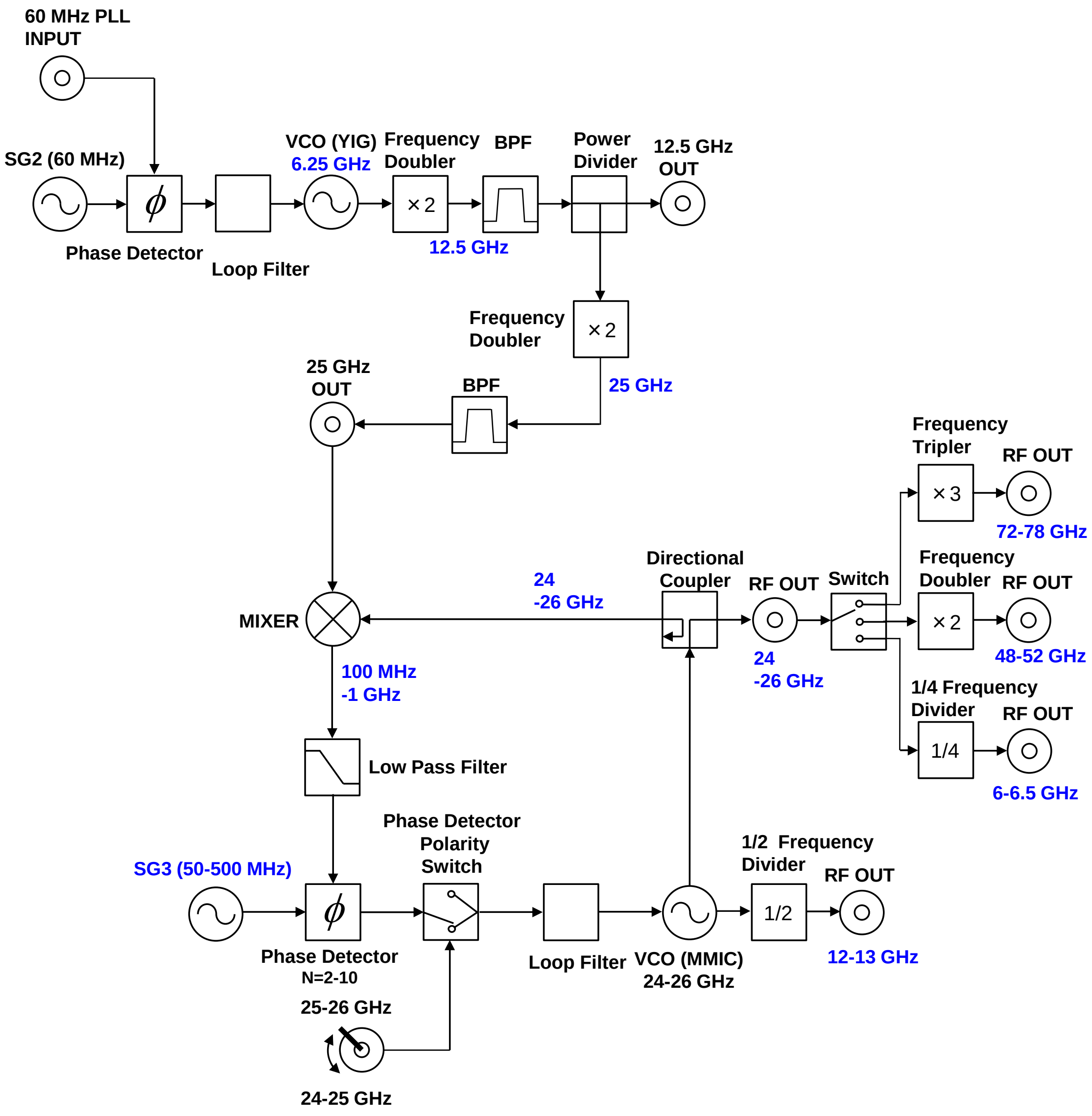

Figure S1. Block diagram of SG 1.

VCO (YIG) is the YIG-oscillator-based VCO, and it generates the low-phase-noise millimetre wave at 25 GHz. VCO (MMIC) is the MMIC-based VCO, and it generates at continuously variable frequencies from 24 to 26 GHz. BPF: Bandpass filter.

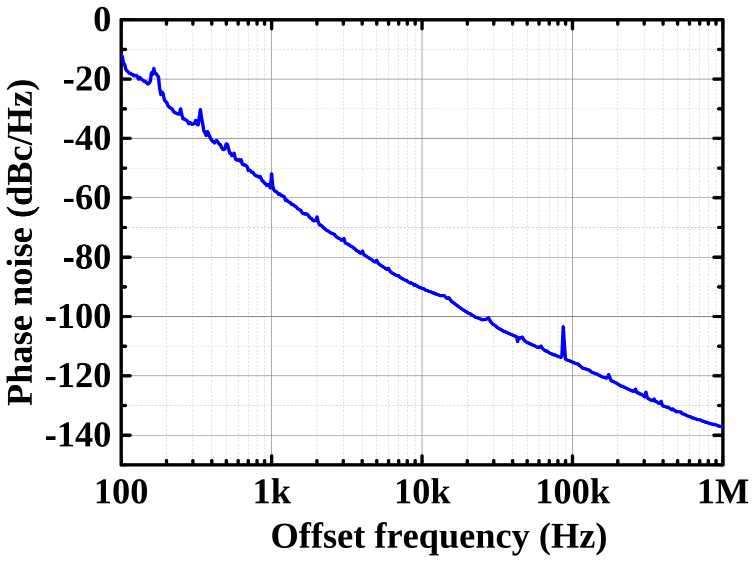

Figure S2. Phase noise of the YIG-oscillator-based VCO at 25 GHz without PLL feedback.

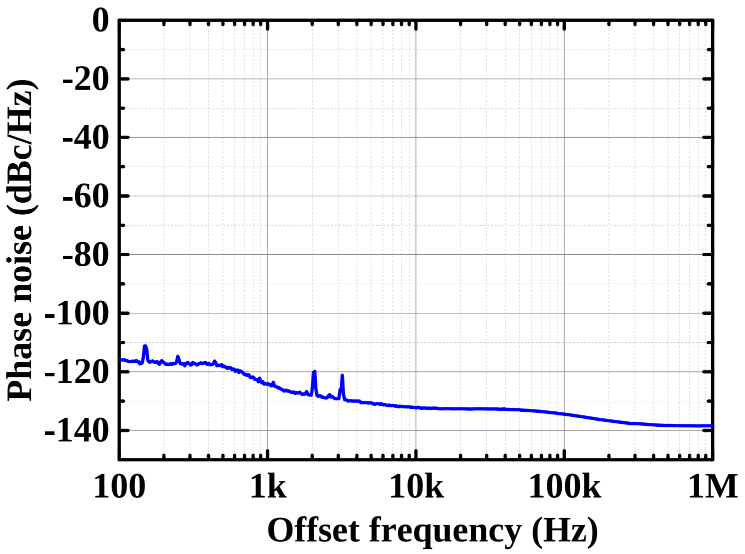

Figure S3. Phase noise of SG 2 at 60 MHz, which is synchronized with the GPS reference signal.

2. Our concept of low-noise millimetre-wave generation

When the CW LD for the seed source of the EOM-OFC is phase/intensity modulated by a sinusoidal signal of frequency f_m, the kth optical field of the EOM-OFC is expressed as

$$E_k(\mathrm{t}) = E_k \exp[-i\{2\pi(f_0 - k \times f_m)t + \varphi_0(t) + k\varphi_1(t)\}]. \qquad \text{(S1)}$$

The optical field of the comb in MLL 1 is expressed as

$$E(t) = E\exp[-i\{2\pi f_n t + \varphi_2(t)\}], \quad \text{(S2)}$$

where f_n and $\varphi_2(\mathrm{t})$ are optical frequency and phase noise, respectively. The combs of MLL 1 are narrowed by using a feedback circuit. Both the kth-mode EOM-OFC and MLL 1 are coupled at the optical coupler and the interference intensity I_{obs} is expressed as

$$I_{obs} = |E_k(t) + E(t)|^2$$

$$= |E_\mathrm{k}(t)|^2 + |E(t)|^2 + 2E_\mathrm{k}E\cos\{2\pi(f_0 - kf_m - f_n)t + (\varphi_0(t) + k\varphi_1(t) - \varphi_2(t))\}.$$

(S3)

The low-frequency component in the interference term I_{int} in eq. (S3) is selected with an RF bandpass filter. The interference term I_{int} is expressed as

$$I_{int}(\mathrm{t}) = V_{obs}\cos\{2\pi\Delta f t + \Delta\varphi\}], \qquad \text{(S4)}$$

where $V_{obs} = 2E_kE,\ \Delta f = f_0 - \mathrm{k} \times f_m - f_n,\ \Delta\varphi = \varphi_0(t) + k\varphi_1(t) - \varphi_2(t).$ SG 2, which is synchronized with the GPS signal, provides the RF reference signal. It generates sinusoidal RF signal I_{ref} with a 90-degree phase shift at frequency Δf, which was set at 60

MHz in our experiment. The RF signal I_{ref} is expressed as

$$I_{ref}(\mathrm{t}) = V_{ref}\sin\{2\pi\Delta f t + \varphi_3(t)\}, \quad \text{(S5)}$$

where V_{ref} and φ_3(t) are the amplitude and phase noise of the RF oscillator, respectively. The interference signal I_{int} is phase-detected with the RF signal I_{ref} inside SG 1. The low-frequency component of the RF signal is then selected with a lowpass filter. The low-frequency component V (t) is expressed as

$$\mathrm{V(t)} = \frac{V_{obs}\times V_{ref}}{2}\sin\{\varphi_0(t) + k\times\varphi_1(t) - \varphi_2(t) - \varphi_3(t)\}. \quad \text{(S6)}$$

The YIG-oscillator-based VCO inside SG 1 is adjusted so that the phase difference becomes zero. Then, the phase noise φ_1 (t) of SG 1 is expressed as

$$\varphi_1(\mathrm{t}) = \frac{\varphi_2(t)+\varphi_3(t)-\varphi_0(t)}{k}. \quad \text{(S7)}$$

Therefore, the phase noise φ_1 (t) of SG 1 decreases as the comb mode number k increases.

3. Supercontinuum generation from EOM-OFC

We produced a short-pulse train at 25 GHz with an intensity and phase modulation method that does not employ a mode-locking technique. The phase and intensity of the light from a CW LD with a centre wavelength of 1552 nm and a linewidth of 800 Hz is sinusoidally modulated at a modulation frequency f_{mod} of 25 GHz from SG 1 with six conventional phase modulators. This creates repetitive up/down-chirp parts at 25 GHz. The linear up-chirp part is picked up by the intensity modulator. The

phase/intensity-modulated laser can generate short optical pulses by compensating for the linear chirp of the up-chirping caused by intensity/phase modulators with a dispersive medium, such as a single-mode fibre. The applied modulation index obtained with the phase modulators is 25π. Our experimental setup yields 150 light carriers with 25-GHz mode spacing. Then the chirped light is amplified to an average power of 1W by the EDFA and compensated with a 1-m-long glass block. With this method, we can generate 186-fs optical pulses (assuming a Lorentzian pulse shape) with a 25-GHz repetition rate. The short optical pulse train is delivered into a 10-m-long highly nonlinear fibre. We successfully demonstrated the generation of SC spectra in the 1450 to 1700 nm range as shown in Fig. S4a. The mode spacing is equal to the 25-GHz repetition rate of the optical pulse train. The 278th comb mode number corresponds to 1611 nm. Figure S4b shows that the 278th comb spectrum has a 30-dB signal-to-noise ratio.

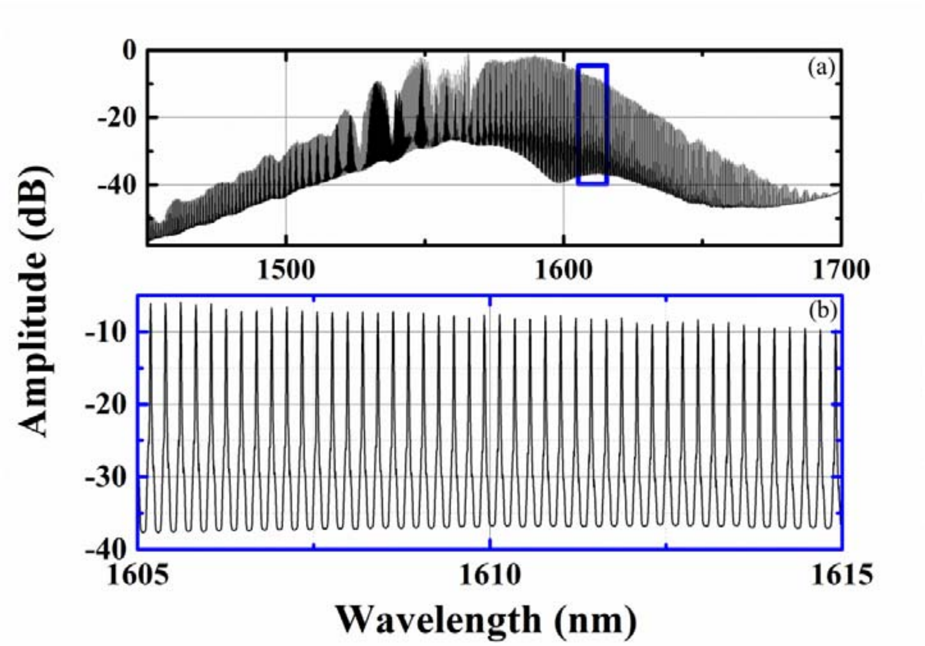

Fig. S4. SC spectrum with EOM-OFC. (**a**) SC spectrum (1450-1700-nm) obtained with a 1-W, 186-fs optical pulse train at 25 GHz. (**b**) SC spectrum near 278th mode number (1611 nm).

4. Measurement of phase noise $\{\varphi_2(t)-\varphi_0(t)\}$

We measure the phase noise $\{\varphi_2(t)-\varphi_0(t)\}$ in eq. (2) with a signal source analyser (E5052B and E5053A) using the interference signal at 60 MHz between MLL 1 and the CW LD. The carrier-envelope offset of MLL 1 is stabilized with an *f*-to-2*f* self-referencing interferometer by using a GPS signal, and the repetition rate is also

locked with the GPS signal and the reference frequency by using the interference signal between the CW LD and MLL 1. The phase-noise spectrum of {φ_2(t) - φ_0(t)} is shown in Fig. S5. We used it to calculate the phase noise of SG 1.

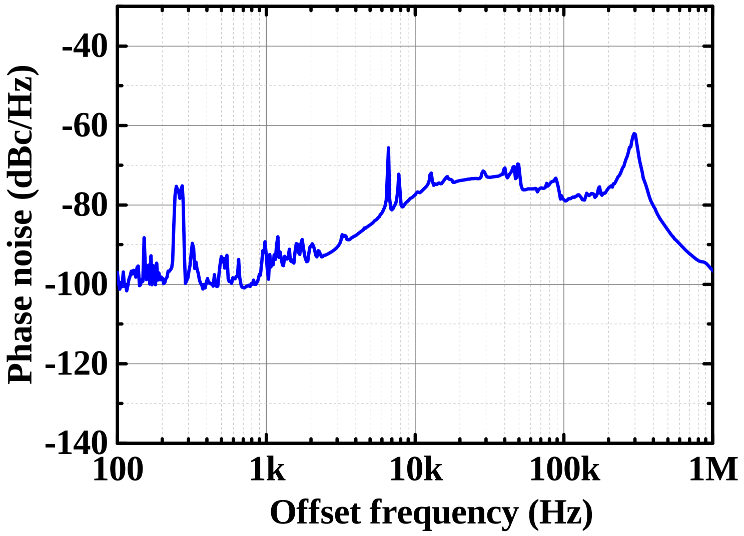

Fig. S5. Phase noise spectrum of {φ_2 (t) - φ_0 (t)}. Phase noise spectrum of {φ_2 (t) - φ_0 (t)} observed from the interference between the CW LD and MLL 1. The phase noise at offset frequencies from 100 Hz to 1 MHz is shown.

5. Relationship between phase-noise φ_1 (t) and CW LD frequency stabilization

We additionally investigated the phase noise φ_1(t) when the centre optical frequency of

the CW LD was stabilized. Figure S6 shows our experimental setup. MLL 2 is used as the reference light source for the frequency stabilization of the CW LD. Both the CEO frequency and repetition frequency of MLL 2 are stabilized. The interference signal between the CW LD and MLL 2 is detected with a photodetector. The centre frequency of the CW LD is stabilized with the feedback circuit by using the interference signal and the GPS signal for the reference signal. The locking bandwidth is several kilohertz. The locking bandwidth is limited by the bandwidth of the feedback circuit used in this experiment. Since the modulation bandwidth of the CW LD is 100 kHz, the locking bandwidth can be increased up to 100 kHz by using a different feedback circuit. Since the linewidth of MLL 2 is around 200 kHz, which is wider than the narrow linewidth comb (800-Hz linewidth in Fig. 1a), the linewidth of the CW LD increases. Figure S7 shows the phase noise for the 278th comb mode number with and without the frequency stabilization of the CW LD. It is very clear that the phase noise at an offset frequency of less than 10 kHz decreases when the centre optical frequency of the CW LD is stabilized. This result shows that, by stabilizing the centre optical frequency of the CW LD while maintaining a narrow linewidth, we should be able to reduce the phase noise at an offset frequency of less than 10 kHz (Fig. 2a). The large phase noise in Fig. S7 is due to the wide linewidth of the CW LD.

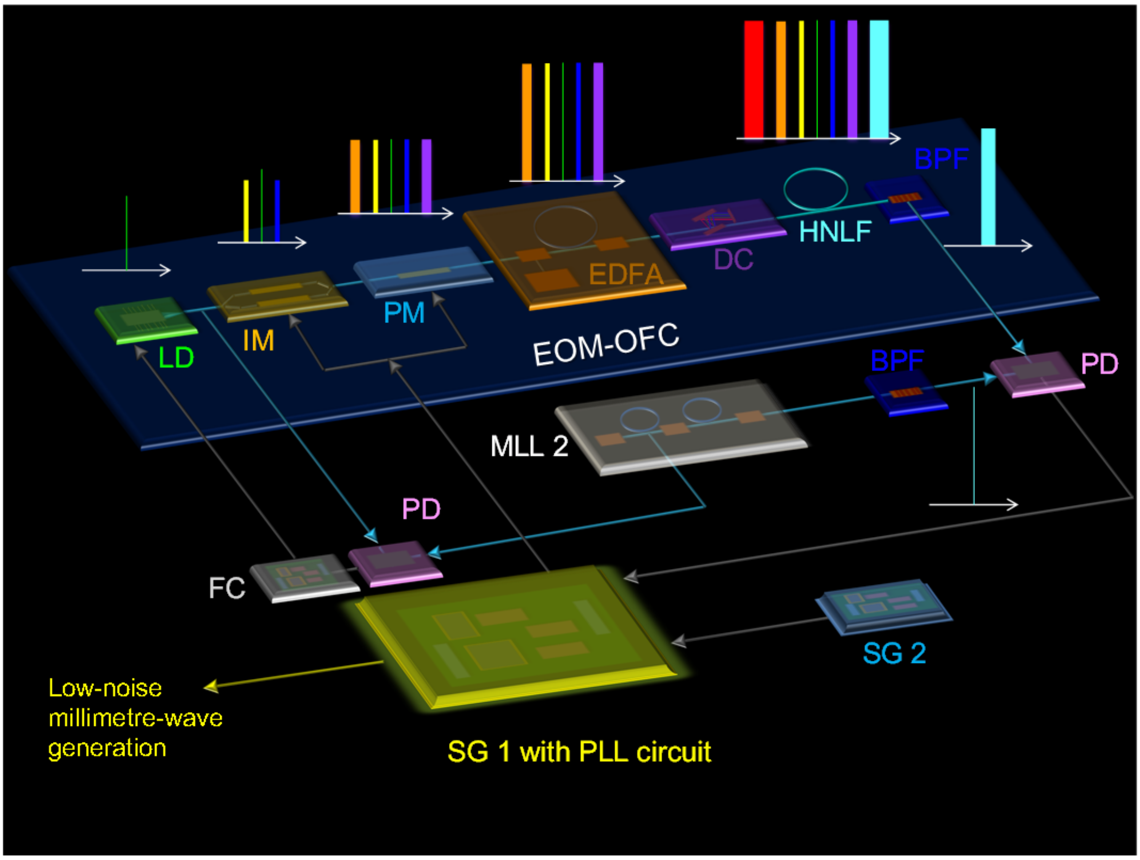

Fig. S6. Schematic diagram of experimental setup when the CW LD centre frequency is stabilized. MLL 2: Mode-locked laser. FC: Feedback circuit.

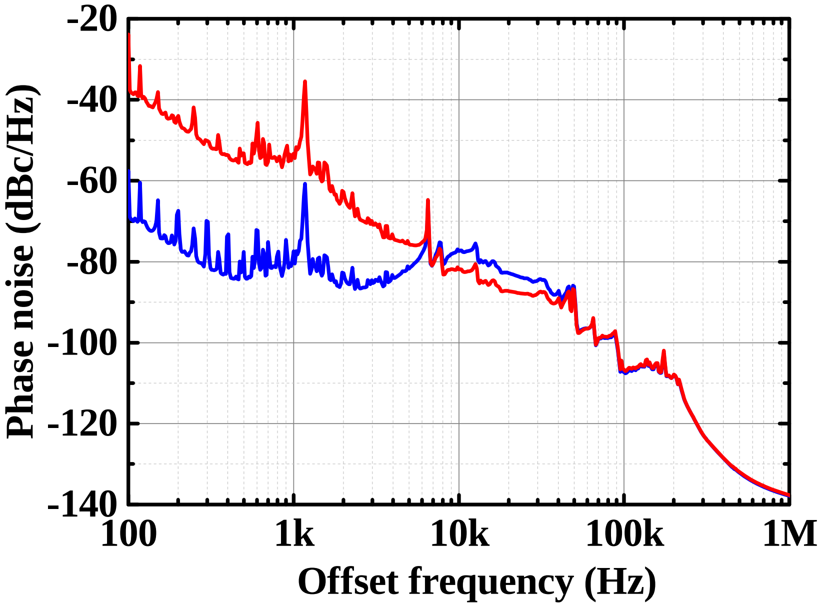

Figure S7. Dependence of phase noise φ_1 (t) of SG 1 on CW LD stabilization. Measured phase noise φ_1 (t) of SG 1 with (blue) and without (red) the optical frequency stabilization of the CW LD at the 278th reference comb mode number.
